# Thoracoscopic thymectomy – The method of choise in surgical treatment of non-invasive thymomas

**DOI:** 10.1016/j.amsu.2018.12.005

**Published:** 2018-12-23

**Authors:** Arif Allakhverdiev, Mikhail Davydov, Goncha Allakhverdieva, Parvin Akhmedov

**Affiliations:** a Department of Surgical Oncology Moscow Clinical Scientific Center, Moscow, Russia Enthusiasts Road 86, 111123, Moscow, Russian Federation; b Department of Thoracic Surgery, N.N. Blokhin Cancer Research Center.Kashirskoe Road 23, 115478, Moscow, Russian Federation; c Ultrasound Department, N.N. Blokhin Cancer Research Center.Kashirskoe Road 23, 115478, Moscow, Russian Federation

**Keywords:** Thoracoscopic thymectomy, Thymoma

## Abstract

**Background:**

Thymomas are very rare malignances, with an estimated incidence of 0.15 cases per 100.000. Metastases of Thymoma are usually involved the pleura, pericardium or diaphragm, whereas the probability of extrathoracic metastases are extremely low. Success in the treatment of thymomas mainly depends on the radicalism of the performed operation.

**Materials and methods:**

Since 2008 33 Thoracoscopic Thymectomies were performed in the thoracic department of the N.N.Blokhin Russian Cancer Research Center. The right-sided approach was in 23 (69,7%) patients, 7 (21,2%) operated on the left and 3 (9,1%) patients had combined approach. We include 26 patients with similar characteristics of the primary tumor operated from sternotomy approach to compare direct and long-term results of surgical treatment.

**Results:**

Thoracoscopic approach in comparison with sternotomy in surgical treatment of non-invasive Thymomas significantly reduces the incidence of postoperative therapeutic complications (p < 0.01). There was a decrease in the frequency of postoperative surgical complications (p = 0.08).

**Conclusion:**

Thoracoscopic thymectomy in comparison with thymectomy from open surgical approach contributes to a significant decrease in the length of patients' stay in the intensive care unit and hospital, and the duration of analgesia with narcotic analgesics in the postoperative period. All of the above factors contribute to shorter periods of functional recovery of patients and a full return to a normal lifestyle.

## Highlights

• Thymomas are very rare, slow growing tumors of the mediastinum.
• Thoracoscopic approach significantly reduces the incidence of postoperative complications.
• Crossing of both Thymus legs.
• Monoblock removal of the surrounding tissue and lymph nodes.

Thymomas are very rare malignances, with an estimated incidence of 0.15 cases per 100.000 [6,9].Thymomas are generally slow growing tumors. Metastases of Thymoma are usually involved the pleura, pericardium or diaphragm, whereas the probability of extrathoracic metastases are extremely low. Success in the treatment of thymomas mainly depends on the radicalism of the performed operation.

Traditionally Thymectomy has been performed from sternotomic [4,5] or trancervical approach [8]. Thoracoscopic technique is widely used for decades with a diagnostic purpose, for differentiating the morphological nature of the mediastinum and allows practically 100% of the observations to achieve verification of the diagnosis [1,2]. Thoracoscopic Thymectomy (TT) was introduced into clinical practice in 1992 [3]. Since the introduction of TT many studies have been conducted, which increased the question of the advantages of the technique in terms of reducing the cost of patient treatment, the length of hospital stay and improving of cosmetic effect. This study is devoted to direct results of surgical treatment of Thymomas using Thoracoscopic technique, as well as the advantages of Thoracoscopic method in comparison with the traditional open surgery.

## 1 Aim of study

The aim of this study is to improve immediate results of surgical treatment of patients with non-invasive Thymomas.

## 2 Materials and methods

In this study we included two groups of patients: patients in the first group were operated via TT, in the second group we performed open Thymectomy. Since 2008 33 TT were performed in the thoracic department of the Russian Cancer Research Center named after N.N.Blokhin. The right-sided approach was in 23 (69, 7%) patients, 7 patients were (21,2%) operated on the left side and 3 (9,1%) patients had combined approach. Combined approach meant a thoracoscpic beginning and mobilization of the tumor (all patients in this group underwent thoracoscopy from the left-side), followed by a patient's turn to the back and sternum dissection. Tumor sizes exceed 10 cm in all patients operated from combined approach. In this case left thoracoscopic approach did not allow safe mobilization of the tumor from the left brachiocephalic vein and mobilization of thymic vein (in view of tumor size). We want to emphasize, the size of tumor is a more significant factor when the tumor is located on the right side than on the left.

The age of patients was ranged from 17 to 71 years and averaged 50, 4 year. The number of operated women was 20 (60, 6%) and prevailed in comparison with men – 13 (39, 4%). From the total number of patients underwent TT morphological type A was diagnosed in 5 (15, 2%) patients, type AB in 10 (30, 3%), type B1 in 7 (21, 2%), type B2 – 8 (24, 2%) patients and 3 (9, 11%) patients with type B3 (Table 1).

**Table 1:** Morphological type of Thymoma in patients underwent Thoracoscpic Thymectomy.

Morphological types	Men	Women	Total
A	3	2	5
AB	3	7	10
B1	3	4	7
B2	4	4	8
B3	–	3	3
Total	13 (39,4%)	20 (60,6%)	33 (100%)

Patient with stage I of the tumor predominated in the group of patients operated from Thoracoscopic approach, their number was 23 (69, 7%). IIA stage was diagnosed in 7 (21, 2%) and 3 patients had IIB stage (9, 1%) (Table 2).

**Table 2:** Tumor type by Masaoka staging system.

Masaoka staging system	Men	Women	Total
T1N0M0	9	14	23
T2aN0M0	3	4	7
T2bN0M0	1	2	3
Total	13 (39,4%)	20 (60,6%)	33 (100%)

In our point of view the maximum tumor size for comfortable and safe surgery by Thoracoscopic approach are tumors up to 7 cm in the largest diameter. In this regard all Thymomas were divided into two groups - tumors larger than 7 a.m. and less. The maximum size of Thymoma, removed thoracoscopicaly, in our study was 12 cm. From the 33 patients who underwent TT the tumor size larger than 7 cm was in 11 (33, 3%) patients and less than 7 cm was in 22 (66,6%) patients. (Table 3).

**Table 3:** Tumor size.

Morphologic type of tumor	Tumor size		Total
	<7 cm	>7 cm	
А	4	1	5
АВ	6	4	10
В1	5	2	7
В2	6	2	8
В3	1	2	3
Total	22(66,7%)	11(33,3%)	33(100%

From total number of 22 patients with tumor sizes less than 7 cm stage I was diagnosed in 15 (68.2%), in 5 patients (22.7%) - stage IIA and in 2 (9.1%) patients IIB stage of Thymoma. Tumors larger than 7 cm were found in 11 patients. The allocation by stages was as follows: Stage I - 8 (72.7%), IIA Stage - 2 (18.2%) and IIB Stage - 1 (9.1%) (Table 4) (see Table 5).

**Table 4:** Patient allocation undergoing thoracoscopic thymectomy according to the tumor size and stage.

Morphologic type of tumor	Tumor size		Total
	До 7см	>7см	
T1N0M0	15	8	23
T2aN0M0	5	2	7
T2bN0M0	2	1	3
Total	22(66,7%)	11(33,3%)	33(100%

**Table 5:** Distribution of patients who underwent thymectomy from open approaches depending on the morphological type and the stage of Thymoma.

Morphological type of tumor	Stage					Total
	T1N0M0	T2aN0M0	T2bN0M0	T3aN0M0	T3bN0M0	
А	6	1	–	–	–	7
АВ	6	–	1	–	–	7
В1	3	–	–	–	–	3
В2	1	4	–	–	–	5
В3	1	–	–	2	1	4
Total	17(65,4%)	5(19,4%)	1(3,8%)	2(7,6%)	1(3,8%)	26(100%)

In terms of preoperative examination for all patients underwent TT we performed computer tomography(CT) to determine the exact location of the tumor, its size and relationship with surrounding organs and structures of the mediastinum. In most cases (90%) we used right-sided thoracoscopic approach. All patients were operated from the so-called “fully thoracoscopic” approach, which involves the use of only thoracoports during the surgery. At the end of the surgery we also did a mini-thoracotomic incision to remove the tumor from the pleural cavity. Its length depends on the size of the removed tumor.

**Fig. 1:**
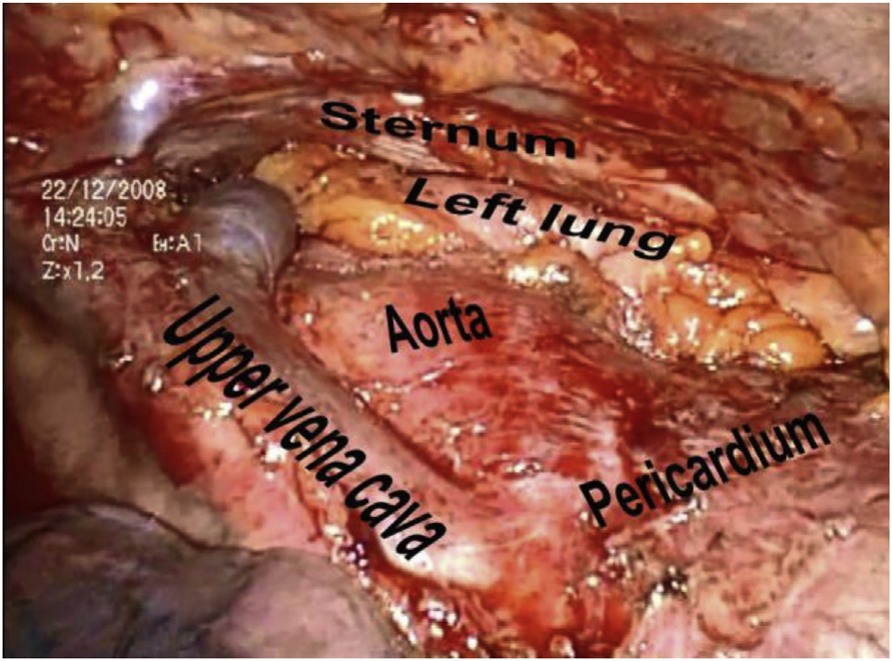
Intraoperative view.

**Fig. 2:**
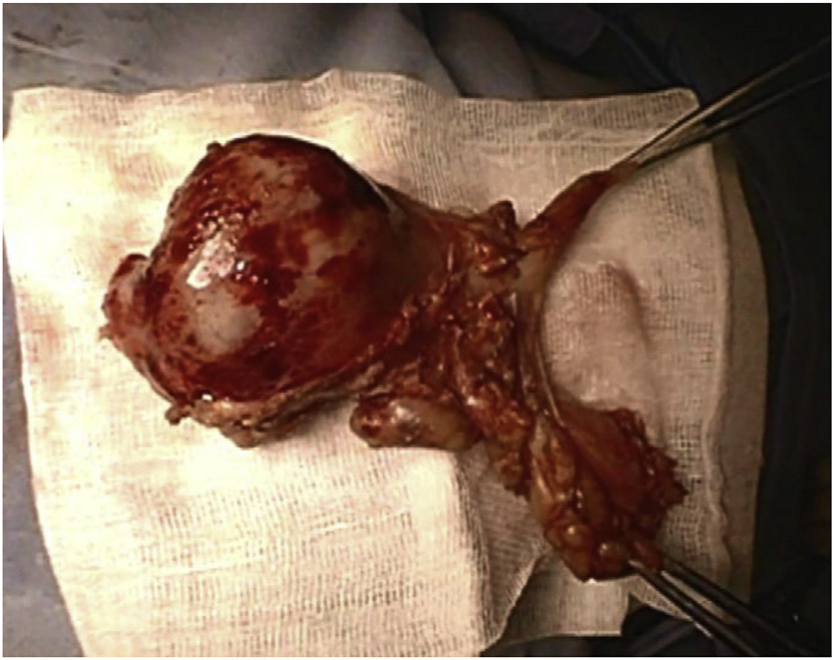
Removed thymoma.

A required condition for TT was a high crossing of both legs of the thymus gland and a monoblock removal of the surrounding tissues and lymph nodes (Fig. 1, Fig. 2).

We include 26 patients with similar characteristics of the primary tumor operated from sternotomy approach to compare immediate and long-term results of surgical treatment of patients with non-invasive Thymus after thoracoscopic and open thymectomy.

Patients with Thymoma type A and AB and I stage of the disease predominated in the group of patients operated from open surgical approaches and accounted for 6 (23.1%) in each group of the total number of patients analyzed in this group. Thymoma B1 was diagnosed in 3 (11,6%) patients, 1 (3,8%) patient with Thymoma B2 and 1 patient with Thymoma B3. Stage II was diagnosed in 1 (3.8%) patient with Thymoma type A, 1 (3.8%) patient with Thymoma AB and in 4 (15.4%) patients with Thymoma type B2. In 3 (11.6%) patients with morphological type B3 of Thymoma III stage was diagnosed.

Depending on the tumor size and the stage of the disease in the open surgery group, patients were distributed as follows: Thymoma A was diagnosed in 5 (19.2%) patients with a tumor less than 7 cm, Thymoma AB was diagnosed in 6 (23.1%) patients, thymoma B1 in 2 (7.6%) patients and only 1 patients with Thymoma B2 (3.9%). In the group of patients with a tumor size more than 7 cm Thymoma A was established in 2 (7.6%), Thymoma AB in 1 (3.9%), thymoma B1 in 1 (3.9%), thymoma B2 was diagnosed in 4 (15, 4%) and thymoma B3-4 (15.4%) of the total number of patients (Table 6).

**Table 6:** Patients who underwent thymectomy from open approaches depending on the size of the primary tumor and Thymoma type.

Morphological type of tumor	Tumor size		Total
	<7см	>7cм	
А	5	2	7
АВ	6	1	7
В1	2	1	3
В2	1	4	5
В3	–	4	4
Total	14(53,8%)	12(46,2%)	26(100%)

In a group of patients who underwent open surgery with tumor sizes less than 7 cm the first stage of the disease prevailed. Total number of these patients is 12 (46.2%).At the same time the second stage was diagnosed in only 2 (7.6%) patients in the open surgery group. In the group of patients with tumor size more than 7 cm stage I was established in 5 patients (19,2%), II stage in 4 (15,4%) and III stage in 3 (11,6%). (Table 7).

**Table 7:** Patients who underwent open thymectomy depending on the stage and size of the tumor.

TNM	Tumor size		Total
	<7см	>7см	
T1N0M0	12	5	17
T2aN0M0	2	3	5
T2bN0M0	–	1	1
T3aN0M0	–	2	2
T3bN0M0	–	1	1
Total	14(53,8%)	12(46,2%)	26(100%)

We use a program Statistica 6.0 to analyze immediate and long-term results.

## 3 Results and discussion

An average time for TT was 2 h. At the same time we spent 2.5–3 h to perform thymectomy in an open surgery group. The total number of postoperative complications in the group of patients who underwent thoracoscopic thymectomy was 6% and was significantly lower in comparison with complications in patients operated from sternotomy approach. In the second group of patients the total complication rate was 46, 1%. The differences obtained are statistically significant (p < 0.01).

The frequency of therapeutic postoperative complications in groups of patients who underwent TT and open thymectomies is presented in Table 8. Comparative analysis of the total number of therapeutic complications showed their significant decrease in the group of patients underwent TT in comparison with patients operated from sternotomy.

**Table 8:** Therapeutic postoperative complications. We also performed a comparative analysis of surgical complications in patients depending on the type of surgery performed. Results are presented in Table 9.

Complications	Access		P
	*Thoracoscopic thymectomy (n* = *33)*	*Open Thymectomy (n* = *26)*	
Pneumonia	1(3%)	2(7,7%)	0,717
Thrombosis	0	3(11,5%)	0,548
Arrhythmias	1(3%)	3(11,5%)	0,572
Sepsis	0	2(7,7%)	0,690
Pulmonary embolism	0	2(7,7%)	0,690
Total	2(6%)	12(46,1%)	0,018

**Table 9:** Comparative analysis of surgical postoperative complications.

Complications	Access		Р
	*Thoracoscopic Thymectomy (n* = *33)*	*Open Thymectomy (n* = *26)*	
Postoperative mortality rate	0	3(11,5%)	0,548
Suppuration of surgical wound	0	2(7,7%)	0,690
Bleeding	0	2(7,7%)	0,690
Total	0	7 (27%)	0,080

The postoperative period was complicated by bleeding in 2 patients (7.1%) operated from open approach. The same number of patients has a suppuration of surgical wound. The fatal outcome was accompanied by 3 surgical interventions performed from sternotomy. Postoperative lethality and surgical complications were absent in the group of patients operated via thoracoscopic approach. The differences were not statistically significant.

We analyzed the total number of surgical complications and found that the number of surgical complications in minimal invasive group was 0% versus 27% in the group of patients operated from open approach. Differences approached the reliability (p = 0.08).

Important criteria in the postoperative period of patients who underwent surgery for tumors of the chest and mediastinal organs are, in addition to postoperative complications, such indicators as the duration of standing of pleural drains, the length of stay of patients in the intensive care unit, using of narcotic analgesics, as well as the hospital stay after the performed surgical access. All these factors influenced the terms of rehabilitation of patients and the restoration of preoperative functions of organs and systems, as well as the timing of postoperative conservative treatment.

**Fig. 3:**
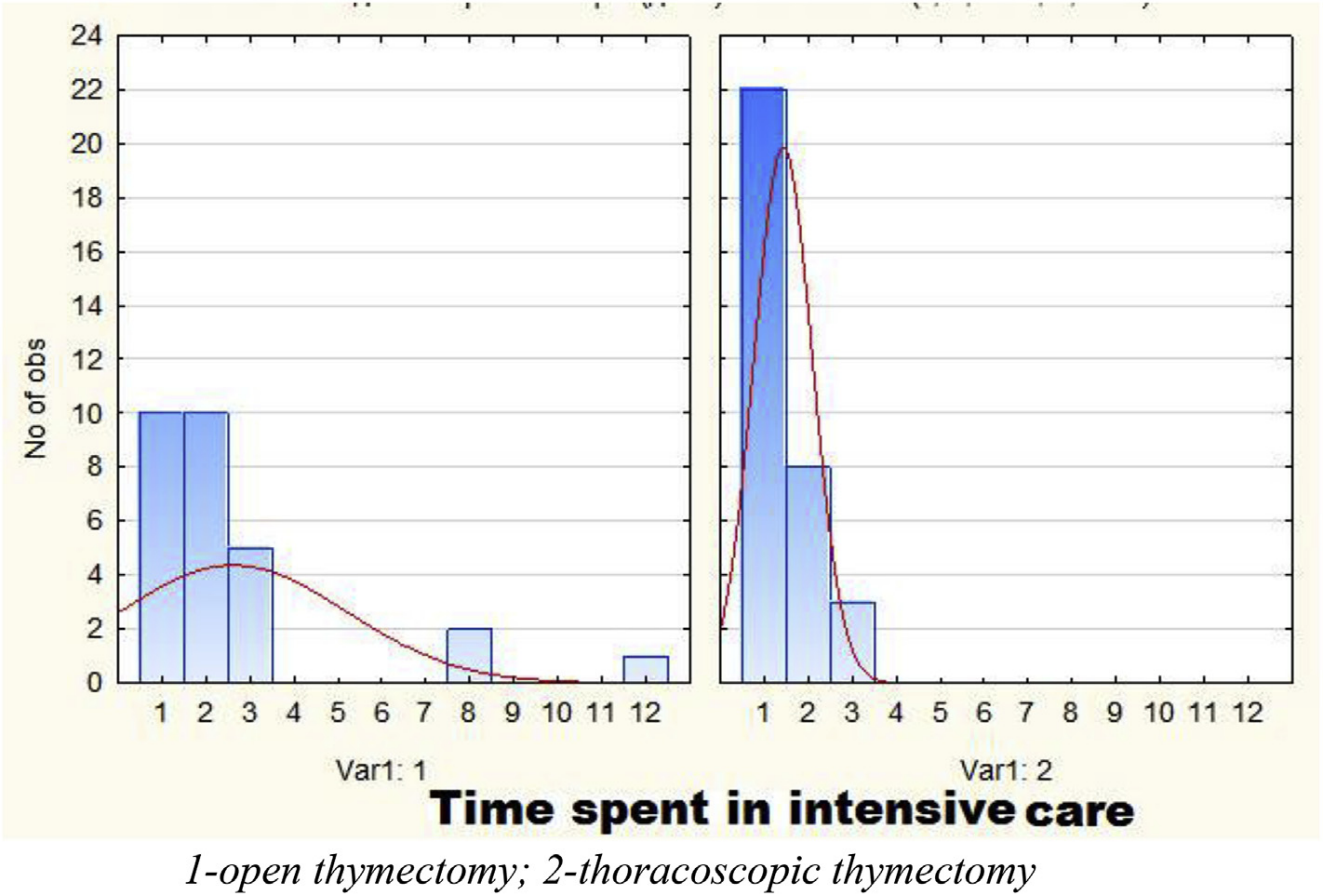
Length of stay in the intensive care unit.

The length of hospital stay in open surgery group was 1–12 days, an average of 2.6 days. In the TT group this indicator averaged 1.4 days and the duration of stay in intensive care department ranged from 1 to 3 days. Comparative analysis revealed a significant decrease in the duration in intensive care department of patients who underwent TT (p = 0.016503). Diagrams of the duration of patients' stay in the intensive care unit are presented in Fig. 3.

**Fig. 4:**
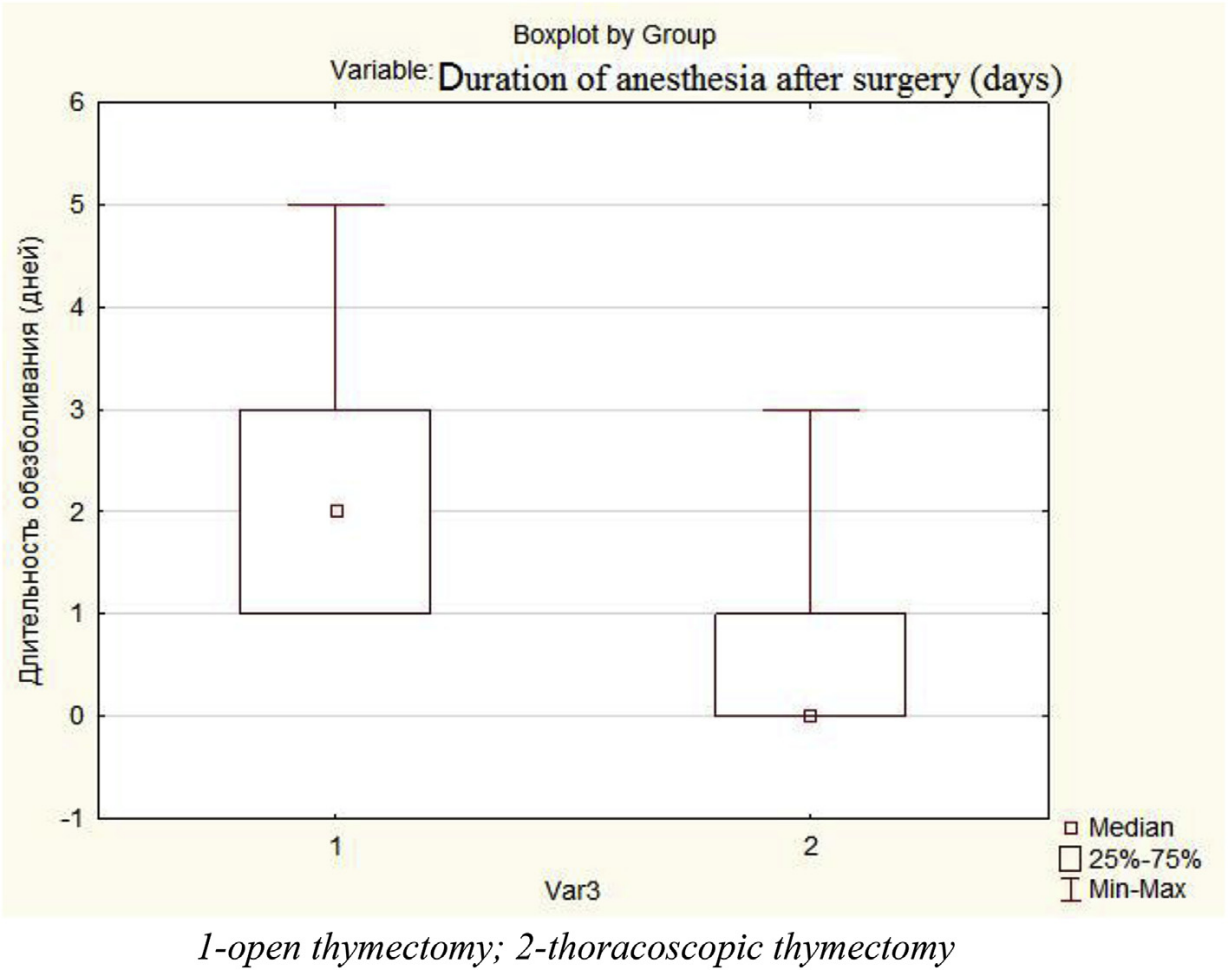
Comparative duration of anesthesia with non-narcotic analgesics after open and thoracoscopic surgeries.

Duration of anesthesia of patients underwent thymectomy is also a very important criteria. We evaluated the features of postoperative analgesia in these two groups of patients. The average duration of anesthesia with narcotic analgesics in TT group was 1.12 days, and was significantly lower in comparison with open thymectomy group. In the second group of patients average of 2.14 days (p < 0.001) was established. A comparison of the data is presented in Fig. 4.

**Fig. 5:**
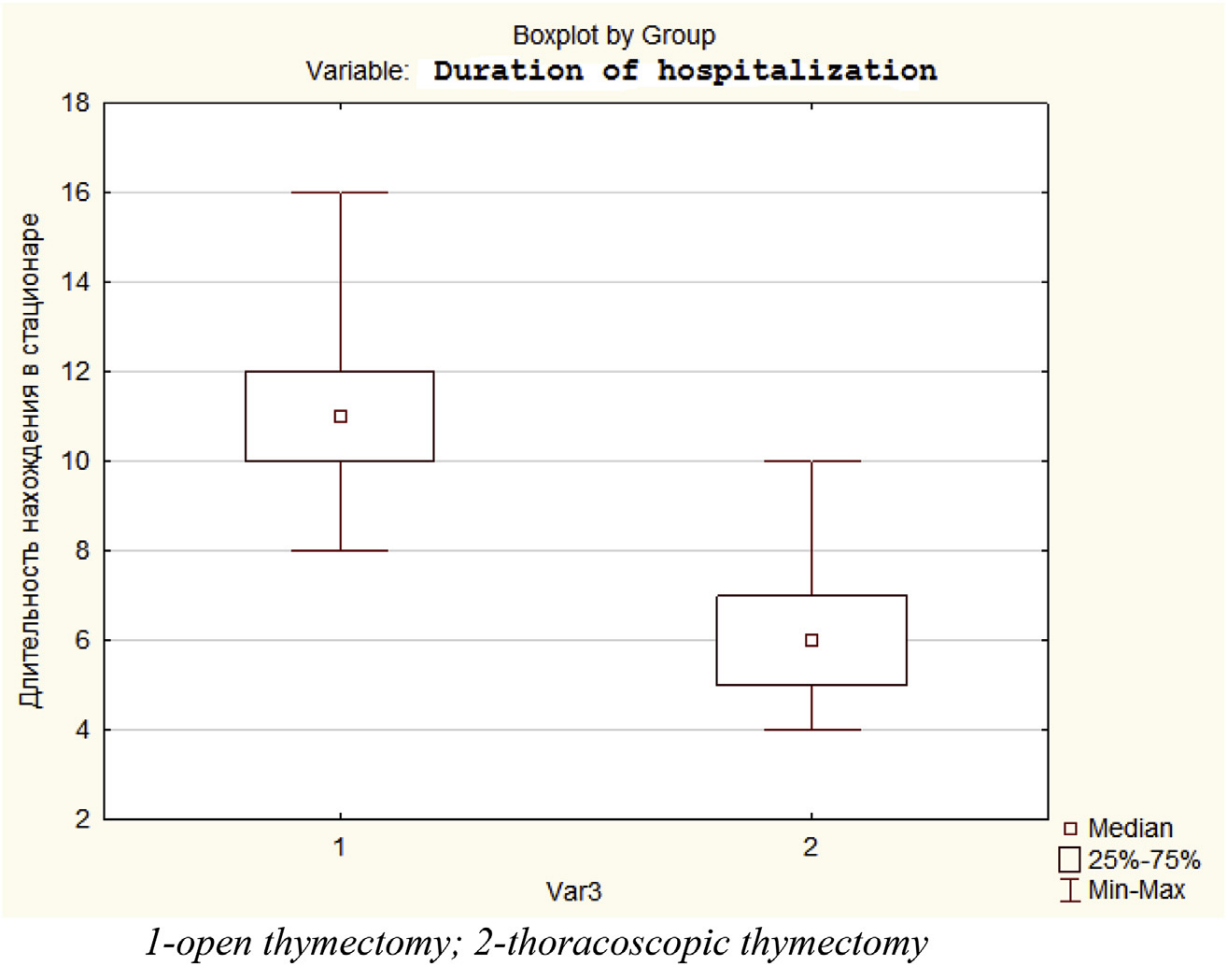
Comparative duration of hospital stay after open and thoracoscopic thymectomies.

The main factors influencing the length of hospital stay were postoperative complications, the duration of healing of the operating wound, as well as the need for postoperative analgesia. A comparative analysis of the duration of hospital stay after the performed thymectomy is presented in Fig. 5.

A statistically significant decrease in the number of hospital stay after surgical intervention was obtained up to 6.2 days after TT from 11 days after open surgery (p < 0.001).

Comparing with a recent meta-analysis, we found that we have no significant differences in such indicators as blood loss (p = 0.004), mean duration of chest tube (as it was lower in TT group) (p = 0.002), hospital stay (p < 0.001) and lower therapeutic and surgical complications (p = 0.03) [11].

## 4 Conclusion

Thoracoscopic approach in comparison with sternotomy in surgical treatment of non-invasive Thymomas significantly reduces the incidence of postoperative therapeutic complications (p < 0.01). There was a decrease in the frequency of postoperative surgical complications (p = 0.08).

TT in comparison with thymectomy from open surgical approach contributes to a significant decrease in the length of patients' stay in the intensive care unit and hospital stay, and the duration of analgesia with narcotic analgesics in the postoperative period. All of the above factors contribute to shorter periods of functional recovery of patients and a full return to a normal lifestyle.

## Ethical approval

Study was exempt from ethical approval from Russian Cancer Research Center N.N. Blokhin.

## Sources of funding

No sources of funding was provided.

## Author contribution

Arif Allakverdiev: Conception of the study, manuscript drafting, and statistical analysis.

Mikhail Davydov: сonception of the study, critical review of the manuscript and supervision.

Goncha Allakhverdieva: interpretation of data and images, analysis and discussion.

Parvin Akhmedov: interpretation of data, critical review of the manuscript, and supervision.

## Conflicts of interest

All the authors declare no conflicts of interest.

## Research registration unique identifying number

researchregistry4189.

## Guarantor

Arif Allakverdiev is guarantor, who accepts full responsibility for the work, has access to the data, and controlled the decision to publish.

## Provenance and peer review

Not commissioned externally peer reviewed.
